# Protein Requirements during Aging

**DOI:** 10.3390/nu8080492

**Published:** 2016-08-11

**Authors:** Glenda Courtney-Martin, Ronald O. Ball, Paul B. Pencharz, Rajavel Elango

**Affiliations:** 1 Research Institute, Hospital for Sick Children, Toronto, ON M5G 1X8, Canada; glenda.courtney-martin@sickkids.ca (G.C.-M.); paul.pencharz@sickkids.ca (P.B.P.); 2 Faculty of Kinesiology and Physical Education, University of Toronto, Toronto, ON M5S 2W6, Canada; 3 Agricultural, Food and Nutritional Science, University of Alberta, Edmonton, AB T6G 2R3, Canada; ron.ball@ualberta.ca; 4 Department of Paediatrics, University of Toronto, Toronto, ON M5G 1X8, Canada; 5 Nutritional Sciences, University of Toronto, Toronto, ON M5S 3E2, Canada; 6 Department of Pediatrics, University of British Columbia, Vancouver, BC V6H 3V4, Canada; 7 Research Institute, BC Children’s Hospital, Vancouver, BC V5Z 4H4, Canada; 8 School of Population and Public Health, University of British Columbia, Vancouver, BC V6T 1Z3, Canada

**Keywords:** elderly, protein, requirements, indicator amino acid oxidation

## Abstract

Protein recommendations for elderly, both men and women, are based on nitrogen balance studies. They are set at 0.66 and 0.8 g/kg/day as the estimated average requirement (EAR) and recommended dietary allowance (RDA), respectively, similar to young adults. This recommendation is based on single linear regression of available nitrogen balance data obtained at test protein intakes close to or below zero balance. Using the indicator amino acid oxidation (IAAO) method, we estimated the protein requirement in young adults and in both elderly men and women to be 0.9 and 1.2 g/kg/day as the EAR and RDA, respectively. This suggests that there is no difference in requirement on a gender basis or on a per kg body weight basis between younger and older adults. The requirement estimates however are ~40% higher than the current protein recommendations on a body weight basis. They are also 40% higher than our estimates in young men when calculated on the basis of fat free mass. Thus, current recommendations may need to be re-assessed. Potential rationale for this difference includes a decreased sensitivity to dietary amino acids and increased insulin resistance in the elderly compared with younger individuals.

## 1. Introduction

Dietary protein plays a key role in various physiological, metabolic, and structural activities of the human body. Therefore, determination of its requirement at every stage of the life cycle requires the application of methods which capture all of the dynamic processes within the human body in which protein participates as total nitrogen and/or amino acids. As science has progressed, a better understanding of the human genome and increased knowledge of human nutrition have revealed that the human body functions best within a range of nutrient intake which is neither too little nor too much. Thus, malnutrition includes both under and over nutrition. The concept of optimal health as it relates to nutrient intake, therefore, refers to a state in which the individual and population intake of nutrients is not only intended to prevent deficiency (minimal intake) but to meet the cells metabolic, physiologic, and structural needs without discrepancy between what is required and what is provided so that the cells equilibrium is maintained (optimal intake). Arguably, aging is one of the stages of the life cycle where the decrease in adaptive capacity suggests a more optimal/ideal nutrient intake will be highly beneficial. 

Aging is accompanied by considerable changes in body composition with increases in fat mass and a progressive loss of skeletal mass [1]. While this is potentially due to several reasons, inadequate protein intake could be a major risk factor [2,3]. Adequate dietary protein could play a key role in preventing this decline in muscle mass [4]. Therefore, there is a keen interest in determining the protein requirements during aging. Over the past 2–3 decades the issue of protein requirements in elderly has been covered in detail by Fukagawa and Young [5], Campbell and Evans [6], Kurpad and Vaz [7], and recently by Nowson and O’Connell [8]. Our objective in this brief review is to describe how the current recommendations for protein in elderly are set, and discuss our recent findings on protein requirements in healthy elderly determined using the stable isotope based-minimally invasive indicator amino acid oxidation (IAAO) method [9]. 

## 2. Current Protein Intake Recommendations 

Current protein intake recommendations for all life stages are set by the Institute of Medicine as the Dietary Reference Intakes (DRI) [10]. The estimated average requirement (EAR) and the recommended dietary allowance (RDA) are set to meet the requirements for 50% and 97.5%, respectively, of a given population. During the last DRI for macronutrient recommendations [10], the protein intake recommendations for elderly men and women were set at 0.66 and 0.8 g/kg/day as the EAR and RDA, respectively. These values were set at the same level as healthy young adults because of the lack of sufficient evidence that aging has an impact on the requirement for protein intake on a body weight basis. 

**Figure 1:**
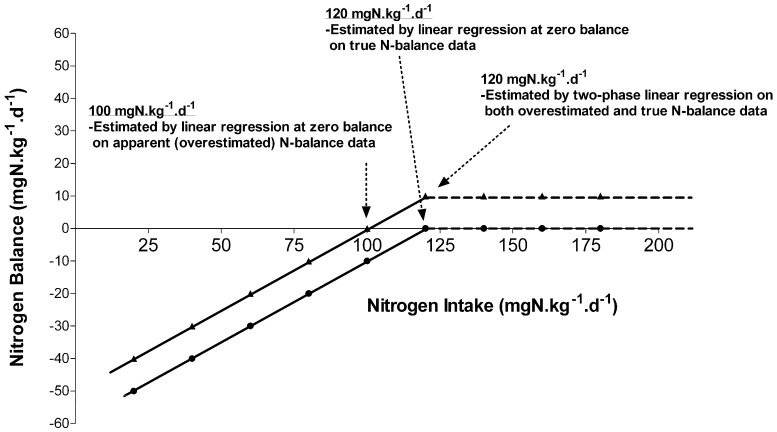
A hypothetical pattern of response in nitrogen balance observed due to increasing intakes of protein. A hypothetical example of the relationship between various protein intake levels and the nitrogen balances (true and apparent, 10% overestimated). Application of linear regression analysis on both true and overestimated nitrogen balance values resulted in nitrogen requirements of 100 and 120 mg/kg/day, respectively (0.63 and 0.75 g/kg/day, protein respectively). Application of the two-phase linear regression analysis on both true and overestimated nitrogen balance values resulted in a nitrogen requirement of 120 g/kg/day. Application of linear regression analysis underestimated nitrogen requirements by 20% when the nitrogen balance values were overestimated by 10%. Adapted with permission from [19].

The current protein requirement recommendations are based on a thorough meta-analysis of available nitrogen balance studies conducted by Rand et al. [11]. The nitrogen balance method has been the traditional method to determine protein requirements. Nitrogen balance identifies the protein requirement in healthy adults as the “continuing intake of dietary protein that is sufficient to achieve body nitrogen equilibrium (zero balance) in an initially healthy person of acceptable body composition at energy balance and under conditions of moderate physical activity and as determined after a brief period of adjustment to a change in test protein intake” [11]. Rand and colleagues acknowledged that there are several shortcomings of the nitrogen balance method, but since there were no other alternative methods at that time, reviewed the data with set criteria. The drawbacks of the nitrogen balance method have been covered in detail by others [12,13,14,15,16]. Briefly, the balance method measures a very small difference in whole body nitrogen intake and excretion and tends to overestimate nitrogen intake and underestimate nitrogen excretion. The net result is an overtly positive balance which could lead to an underestimation of the requirement [10]. In most adult studies, positive balances are often obtained, although it is biologically not possible to be achieved. Balance studies also require relatively long periods of test diet adaptation (7–10 days), because equilibration of the slow changing and large body urea pool requires at least 5–7 days due to a change in test protein intake [17]. Nitrogen balance is also affected by energy intakes, and several of the earlier studies were conducted with higher energy intakes [10]. The final and major points to be discussed are data analysis considerations of the nitrogen balance data, because the efficiency of nitrogen utilization decreases near zero balance [18], and the relationship between intake and balance is curvilinear. Thus, with increasing nitrogen intake the nitrogen response curve is non-linear. Unfortunately, almost all the earlier balance studies provided test protein intakes at or near zero balance. Thus, the intercept, usually determined by linear interpolation, leads to an underestimation of the true balance, as argued by us (Figure 1) [19]. This was evident in the meta-analysis, in which a single linear regression analysis was applied to 19 studies which included nitrogen balance responses from 235 subjects, tested at ≥3 test protein intakes, and was used to derive the current protein recommendation [10]. Rand and colleagues did perform other statistical model analysis on the data set, including log, asymptotic exponential, and biphase linear regression analysis, and reported that all these models provided higher estimated nitrogen requirements than their primary linear regression analysis [11]. 

Based on the above discussion, we re-analyzed the nitrogen balance studies using biphase linear regression analysis and, in addition to the 19 studies in the original analysis, we included an additional 9 studies which met inclusion criteria [20]. Our selection criteria were equally robust, i.e., repeated measures within the same subject, with ≥3 test protein intakes, adaptation to test intake for ≥6 days, and use of standard nitrogen balance techniques. In particular, our reanalysis included test intakes above the intercept of where the zero balance occurs, and our reanalysis resulted in an EAR and RDA for protein of 0.91 and 1.0 g/kg/day, respectively [20]. 

Subsequent to the DRI report [10], the World Health Organization/Food and Agricultural Organization/United Nations University (WHO/FAO/UNU) which sets protein intake recommendations worldwide, published their report [21]. In essence, the same data set was utilized by the WHO/FAO/UNU and the average and population safe protein intake recommendations for human adults (young and old) were set as 0.66 and 0.83 g/kg/day, respectively. The WHO/FAO/UNU, in keeping with the DRI committee’s assessment, concluded that based on the limited dataset available there are no age related changes in protein requirements [21]. 

In 2008, Campbell and colleagues published what was quite possibly the largest comparison of protein requirements between young and elderly adults using the nitrogen balance method [22]. Twenty-three younger men and women were compared against nineteen older men and women across test protein intakes of 0.5, 0.75 and 1 g/kg/day. The mean requirement determined using linear regression analysis was not different between younger and older individuals (0.61 ± 0.14 and 0.58 ± 0.12 g/kg/day, respectively). While this study provides evidence that there are likely no differences in protein requirements between young and elderly humans on a body weight basis, there are several limitations, which must be addressed. The range of test intakes studied (with the highest being 1 g/kg/day), are not wide enough to estimate a true requirement. In addition, the statistical test (single linear regression) applied to the data does not take into account the physiological aspects of protein metabolism and the inherent underestimation of requirements due to the nitrogen balance method; these factors make it impossible to accurately derive an estimate of the protein requirement using the data from that important study. 

Thus, as recommended by the WHO/FAO/UNU report [21], there was and is an urgent need to develop alternative methods to determine protein requirements in humans. 

## 3. Protein Requirements Determined by Indicator Amino Acid Oxidation 

**Figure 2:**
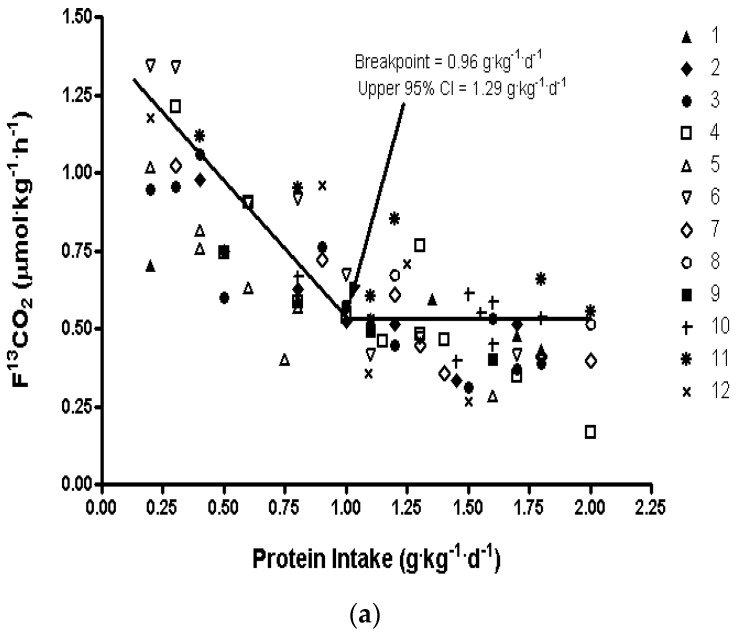

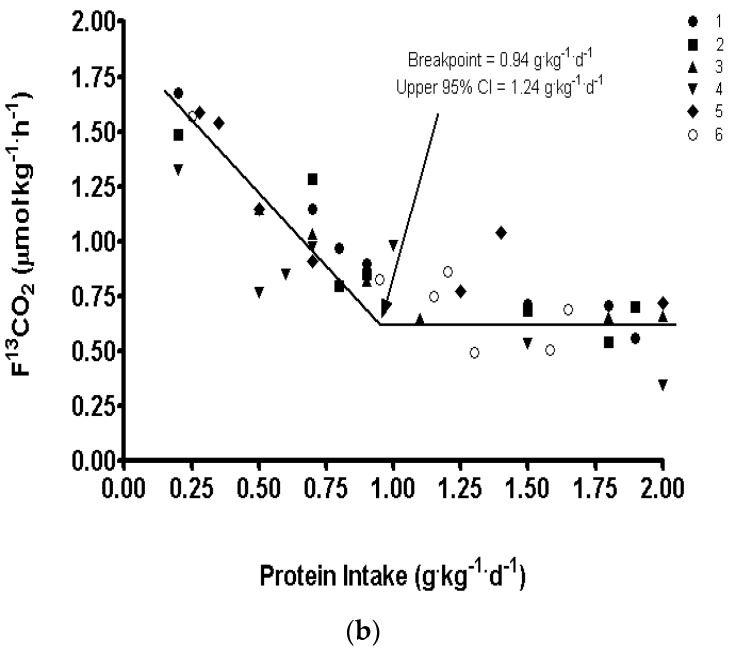
Protein requirements in healthy elderly determined using the IAAO method (**a**) Women >65 years, *n* = 12, each subject participated in a minimum of two test intakes for a total of 82 studies, mean (population safe intakes) = 0.96 (1.29) g/kg/day; (**b**) Men >65 years, *n* = 6, each subject participated in seven test intakes for a total of 42 studies, mean (population safe intakes = 0.94 (1.24) g/kg/day.

The indicator amino acid oxidation (IAAO) method was originally developed for the determination of individual amino acid requirements in baby pigs [23]. Over the past two decades we have utilized the method to determine amino acid requirements in healthy and diseased adults, children, and in neonates [9,24,25,26]. The results from studies in healthy young men were used by the Institute of Medicine to set amino acid requirement recommendations for North Americans [10]. Most notably, amino acid requirement estimates from published IAAO studies, reviewed earlier by Elango et al. [10], were used by the DRI committee to set recommendations for lysine, methionine, phenylalanine, threonine, and tryptophan [10]. The fundamental concept of the method is that with decreased intake of a limiting (test) amino acid, oxidation of all other amino acids, including an indicator amino acid (another indispensable amino acid labeled with a stable isotope, usually 1-^13^C-Phenylalanine) will be oxidized, as there are no large stores of amino acids in the body. With increasing intakes of the test amino acid, oxidation of the indicator amino acid will decrease, reflecting the increasing incorporation of the test amino acid towards protein synthesis. Once the requirement for the test amino acid is reached, oxidation of the indicator amino acid will plateau (Figure 2a,b). The inflection/breakpoint where the oxidation plateaus, identified using biphase linear regression analysis, determines the mean requirement or the EAR, and the upper 95% CI determines the RDA or population safe requirement. 

The method has been subsequently adapted over the years to be minimally invasive; such that the delivery of isotope is oral rather than intravenous, subjects are adapted to an adequate protein intake for two days only, and only adapted to the test amino acid intake on the study day. In addition, measurement of label tracer oxidation is done in breath and urine rather than blood [24]. Thus, the method is well suited for vulnerable populations, including elderly. We reasoned that the IAAO method could be applied for the determination of protein requirements, and conducted our first study in young adult men aged (~29 years) [20]. Using 1-^13^C-Phenylalanine as the indicator, we measured its oxidation in breath in response to graded intakes of protein (range 0.1–1.8 g/kg/day). With increasing test protein intake the oxidation of 1-^13^C-Phenylalanine decreased up to a point, then plateaued. Using biphase-linear regression crossover analysis we identified a breakpoint (reflecting the mean requirement estimate or EAR) at 0.93 g/kg/day, and an upper 95% confidence interval (CI) (reflecting the RDA estimate) of 1.2 g/kg/day (Table 1). These results are identical to our re-analysis of the nitrogen balance studies (presented in the same report), which determined the protein requirements to be 0.91 and 1.0 g/kg/day [20]. 

Having validated the minimally invasive IAAO method in young, healthy adult males for the study of protein requirement, we applied the method to the evaluation of the protein requirement in a more vulnerable group: women aged 80 years and above by way of our collaboration with Campbell and colleagues [27]. Six women participated in the study with an average age of 82 years. Using a similar protocol to that employed in the study in young men, each older woman received one of seven test protein intakes in random order ranging from 0.1 to 1.8 g/kg/day. A breakpoint in IAAO was determined to be 0.85 g/kg/day and the upper 95% CI of the estimate was 1.15 g/kg/day [27]. These requirement estimates are similar to those derived in young, healthy men, (Table 1), but together they suggest that the current protein recommendation published by the DRI and FAO for adults (including elderly) are underestimated by ~30%. These results are also in agreement with those published by Campbell et al. using the nitrogen balance method [22] that on a body weight basis there does not appear to be an increase in protein requirements with aging. 

The report by Tang et al. [27] presented data which showed that protein requirements of women aged 80 years and above were higher than the current recommendations. However, the sample size being relatively small (6 women, 42 studies) resulted in very large confidence intervals in the biphase linear regression analysis. Thus, we conducted a much larger protein requirement study in elderly women (*n* = 12), ranging in age from 65 to 85 years using the same IAAO protocol [28]. The women in this study had an average age of ~74 years, and received test intakes ranging from 0.2 to 2.0 g/kg/day. The breakpoint and the upper 95% CI of the estimate were determined to be 0.96 and 1.29 g/kg/day (Table 1, Figure 1a), and these results are ~40%–60% higher than current recommendations. We also recently applied the same IAAO method in elderly men 65 years and above [29]. In this study, six men ranging in age from 66–79 years received test intakes ranging from 0.2 to 2.0 g/kg/day The mean and upper 95% CI of the estimate were determined to be 0.94 and 1.24 g/kg/day, respectively (Table 1, Figure 1b). Thus, our estimates of protein requirements in young adults [20] and elderly men [29] and women [27,28] suggest that when assessed purely on the basis of body weight, aging does not have a measurable effect on protein requirement. However, our results consistently demonstrate that current recommendations for protein intake in adults, both young and old, are underestimated. 

Limitations of the IAAO method have been discussed [10]. Key issues are that that the method has only been used in the fed state, diets are amino acid based, and doubts about whether the indicator amino acid could be limiting and impact the determined protein requirements, since it is held constant. But, the protein requirement estimate derived using the IAAO method was the same (0.93 g/kg/day) as that derived from nitrogen balance studies to which a biphase linear regression analysis was applied (0.91 g/kg/day) [20]. The nitrogen balance studies include the use of food-based diets and include 24 h fed and fasted cycles. In the IAAO studies, we use Phenylalanine at the level of requirement and in the presence of adequate tyrosine, which ensures that the indicator amino acid is never limiting in the test diet and does not affect protein requirements. And in all our IAAO studies, phenylalanine flux remains unaffected at all test protein intakes and provides additional evidence that the indicator amino acid pool remains stable, and the resulting IAAO reflects whole body protein synthesis. 

## 4. Expression of Protein Requirements on a Bodyweight or Fat Free Mass Basis 

Protein requirement estimates and prescription provided on a body weight basis are important, because, for practical purposes, public health recommendations are made on a body weight basis. However, recommendations made on body weight basis fail to acknowledge the difference in body composition between younger and older adults. In our protein requirement study conducted in young men [20], older men [29], and older women [28], the mean fat free mass as a percentage of body weight was 81%, 59%, and 60% in young men, old men, and old women respectively. This significantly higher fat free mass in the young men, translated into a protein requirement of 15% of resting energy expenditure in the younger, compared to the 21% in the older male and female adults. Also, calculated on the basis of fat free mass, the protein requirement estimate of the elderly male and female subjects is 1.6 g/kg/day compared with 1.1 g/kg/day in young adult men (Table 1). This means that relative to calorie intake, the diet of elderly persons should be denser in proteins compared with younger adults. A prescription made solely on the basis of body weight in elderly persons, sends the message that a 75 kg elderly human has a similar metabolism to a 75 kg 25-year-old healthy adult human.

Aging is associated with a decrease in resting energy expenditure as a result of loss of lean body mass and a decrease in the contribution of muscle compared to a relative increase in non-muscle lean tissue to whole-body protein metabolism [30,31]. Loss of lean muscle mass (sarcopenia) is multifactorial; it is associated with sedentary lifestyle [32], increase in abnormal reactive oxygen species (ROS) and glutathione deficiency [33], as well as insulin resistance and abnormal mitochondrial function [34]. Additionally, aging is associated with a loss of sensitivity of skeletal muscle to protein ingestion [35,36] and an increased sequestration of amino acid in splanchinc tissue resulting in a net decrease in peripherally available amino acids for muscle protein synthesis [37]. This is important because the main stimulators of muscle protein synthesis as well as regulators of protein breakdown are amino acids [38,39].

There is no difference in skeletal muscle turnover in the post-absorptive state between young and elderly [40]. In addition, aging does not impair muscle protein synthesis in response to the intake of relatively large amounts of protein (30 or 113 g) [41,42] or essential amino acids (15 g) [43]. However, when protein [35] or amino acids [44] are given in smaller amounts, protein synthesis is blunted in the elderly relative to the young. Despite differences in diets across cultures, in general, foods are eaten as mixed meals in which carbohydrate provides the main source of energy in the diet. Therefore, the study of Volpi et al. [45] is significant. In that study the ingestion of an amino acid-glucose mixture produced a blunted response in the elderly whereas muscle protein synthesis increased in the young. The suggestion is that aging is associated with a decreased sensitivity to amino acid stimulus in the context of a carbohydrate containing mixed meal [46]. This could in part explain the higher protein requirement observed per kilogram of fat free mass in our studies of protein requirement in elderly men and women [28,29]. 

The observed decreased sensitivity of skeletal muscle of older adults has led Paddon-Jones and colleagues to suggest that a moderate serving of high biological value protein (30 g; ~12 g essential amino acids) be added to each meal consumed by the elderly [46]. This pattern of eating will provide about 90 g of protein per day or 1.2 g/kg body weight to a 75 kg elderly person. This is in keeping with observations that aging is associated with a decreased response of skeletal muscle to low doses of essential amino acids [44] but that intake greater than 30 g of protein does not produce a greater anabolic response in the elderly [41].

Protein pulse feeding, in which 72% of the total daily dietary protein (provided at 1.31 g/kg/day) was provided at the noon meal to elderly persons for 6 weeks, resulted in an increase in lean mass index, appendicular skeletal muscle mass, and body cell mass when compared with adults who received their protein spread over the day as 4 meals [47]. The positive effect on lean mass was explained by the increased plasma essential amino acid concentration observed after the pulse relative to the even spread feeding [48]. These studies suggest that although elderly persons have a decreased muscle mass, they require an increased stimulus driven by the presence of a higher intracellular concentration of amino acids to obtain similar rates of protein synthesis. This points to a greater requirement per kilogram of fat free mass and further supports the finding from our studies.

The impact of digestion and absorption kinetics on type and amount of protein intake on muscle protein synthesis has been tested in elderly subjects in response to varying whey protein intakes of 10, 20, and 35 g on its own [49] and in response to casein intake of 20 g eaten alone or in combination with carbohydrates [50]. A linear increase in muscle protein synthesis was observed in response to whey protein intakes with the lowest and highest synthesis observed at intake of 10 and 35 g of whey protein, respectively [49]. The intake of 20 g casein combined with carbohydrate in young and old subjects resulted in similar rates of muscle protein synthesis after 5 h in both older and younger subjects. Additionally, the 20 g casein resulted in similar muscle fractional synthesis rates [50] as that observed after consuming the highest intake of whey (35 g) [49]. These data provide evidence that the type of protein has an effect on post-prandial muscle protein synthesis in elderly subjects but, regardless of type, a higher intake of protein of at least 20 g per meal is needed to stimulate muscle protein synthesis in older compared with younger individuals. Indeed, recently Loenneke et al. [51] using the NHANES 1999–2002 data on food intake in adults 50–85 years found that frequent consumption of meals containing 30 to 45 g protein per meal was associated with leg lean mass and strength. 

The suggestion is that while the current DRI recommendations for protein might be adequate to promote nitrogen balance in healthy elderly individuals, it is inadequate to promote optimal health and prevent progressive loss of muscle mass. 

## 5. Functional Evidence for a Higher than Current Protein Recommendations in Elderly 

Although it is understood that population based studies of protein intake and its relationship to clinical outcomes are difficult to interpret, the inherent difficulty in conducting long-term metabolic studies in which protein intake is appropriately controlled has led to a strong reliance on data generated from retrospectively collected data. Data from Houston et al. [52] in which they assessed the dietary intake of 2066 men and women aged 70–79 years showed that subjects in the highest quintile of protein intake (1.1 g/kg/day/18.2% of calories) lost 40% less lean mass over the course of three years than those in the lowest quintile of intake (0.7 g/kg/day/12.7% of calories). Recent data from the Framingham Offspring Cohort study [53] (men = 1166, women = 1509) showed that in both men and women, leg lean mass was higher and quadriceps strength was higher in participants in the highest quartile of protein intake compared with those in the lowest quartile. For men and women, the lowest quartile of protein intake represented 0.7 and 0.8 g/kg/day whereas the highest quartile represented 1.2 and 1.32 g/kg/day respectively. In both studies the lowest protein intakes, which resulted in the poorest outcomes, are closest to the current DRI recommendations, whereas the highest intakes, with the most positive outcomes, mimics the requirement estimates derived by our group using the IAAO method. 

Recently Tieland et al. [54,55] published two randomized double-blind placebo controlled trials of protein supplementation for 24 weeks in frail elderly. Protein supplements (~15 g, twice a day) increased protein intake in the test group to 1.3 to 1.4 g/kg/day, and led to no change in muscle mass, but led to increased muscle strength, in particular, leg extension strength and improved physical performance (measured by balance, gait, and chair lift ability) [55]. In addition, it was shown that resistance exercise for 24 weeks indeed improved strength and physical performance with an increase in muscle mass in frail elderly, but the additional protein intake (~1.4 g/kg/day) was required to gain the increase in muscle mass [54]. Numerous studies have also shown a beneficial effect of protein intake on bones at levels above the current recommendations. Positive effects on bone mineral density [56] and hip fractures [57] have been reported. In addition, hypercalcemia observed with increasing protein intake up to 2.0 g/kg/day was due to increased intestinal absorption of calcium with the high protein intake rather than a negative effect of protein on bone health [58,59]. Misconceptions exist regarding the effect of high protein on bone and renal health. While it is currently accepted that a high protein diet is harmful to individuals with existing kidney dysfunction, a protein intake at the level estimated by our group for older adults has not been found to be dangerous to healthy individuals [60,61]. 

Current evidence suggest that aging is associated with glutathione deficiency, which results in increased oxidative stress [33], impaired fasted oxidation of nonessential fatty acids, and insulin resistance [62]. Young adults fed a protein intake based on the 1985 FAO/WHO recommendation of 0.75 g/kg/day experienced a glutathione deficiency compared to that observed when they consumed their habitual diet in which protein was consumed at 1.13 g/kg/day [63]. In that study, young adults were able to maintain nitrogen balance despite the antioxidant deficient state [63]. In the elderly, where decreased sensitivity to dietary protein requires increased protein and/or essential amino acids to stimulate the same rate of muscle protein synthesis as that observed in younger persons, it raises the question whether current recommendations for protein are sufficient to maintain glutathione synthesis. Furthermore, it was shown recently in elderly that glutathione deficiency was corrected when the diet was supplemented with the glutathione precursors cysteine and glycine [33]. Since cysteine is a non-essential amino acid synthesized from the essential amino acid methionine, and hence the rate limiting amino acid for glutathione synthesis, this begs the question as to whether the underlying problem is one of dietary protein inadequacy or amino acid inadequacy. This is relevant because we have shown that when adequate protein and methionine are provided to young adults, supplemental cysteine does not increase glutathione synthesis [64]. Nevertheless, in the elderly, it is possible that a glutathione deficiency could reflect a protein deficiency and/or a deficiency of specific amino acids. Thus, in the future, there exists a need to determine optimal amino acid needs in elderly to optimize protein nutrition.

## 6. Conclusions 

In conclusion, the current recommendations for protein intake in elderly based on the nitrogen balance data are potentially underestimates. The IAAO method presents an alternative to nitrogen balance for the derivation of dietary protein requirements. The estimates for protein requirements in both elderly men and women were derived to be 0.9 and 1.2 g/kg/day as the EAR and RDA, respectively. The higher derived estimate is similar to the amount of dietary protein required for better physiologic, metabolic, and functional capacity as assessed from metabolic, epidemiological, and randomized controlled studies, as described above. The efficacy of the derived estimate in promoting sustained health and functional well-being in elderly needs to be tested in future long-term prospective clinical studies. 

## Figures and Tables

**Table 1:** Protein requirements in adults.

	Estimated Average Requirement, EAR (g/kg/Day)	Recommended Dietary Allowance, RDA (g/kg/Day)	Estimated Average Requirement, EAR (g/kg Fat Free Mass (FFM)/Day)	% of Total Calories (Resting Energy Expenditure (REE) × 1.7)
**Current protein recommendations for young and older adults**				
Adult DRI’s ^1^	0.66	0.8		-
**Data from studies in young adults**				
Re-analysis of N Balance ^2^	0.91	1.0		-
IAAO, young men	0.93	1.2	1.14 ± 0.09	10%–13%
**Data from studies in older male and female adults**				
IAAO, 80–87 years women	0.85	1.15	-	10%–13%
IAAO, 65–85 years women	0.96	1.29	1.62 ± 0.14	13%–15%
IAAO, 66–79 years men	0.94	1.24	1.59 ± 0.15	14%–18%

^1^ Current recommendations [10], based on single linear regression analysis of Nitrogen balance data; ^2^ Reanalysis of Nitrogen balance data using two-phase linear regression analysis; IAAO, indicator amino acid oxidation.

## References

[1] Cruz-Jentoft A.J., Landi F., Schneider S.M., Zuniga C., Arai H., Boirie Y., Chen L.K., Fielding R.A., Martin F.C., Michel J.P. (2014). Prevalence of and interventions for sarcopenia in ageing adults: A systematic review. Report of the International Sarcopenia Initiative (EWGSOP and IWGS). Age Ageing.

[2] Morley J.E. (2015). Nutritional supplementation and sarcopenia: The evidence grows. J. Am. Med. Dir. Assoc..

[3] Volpi E., Campbell W.W., Dwyer J.T., Johnson M.A., Jensen G.L., Morley J.E., Wolfe R.R. (2012). Is the optimal level of protein intake for older adults greater than the recommended dietary allowance?. J. Gerontol. A Biol. Sci. Med. Sci..

[4] Paddon-Jones D., Short K.R., Campbell W.W., Volpi E., Wolfe R.R. (2008). Role of dietary protein in the sarcopenia of aging. Am. J. Clin. Nutr..

[5] Fukagawa N.K., Young V.R. (1987). Protein and amino acid metabolism and requirements in older persons. Clin. Geriatr. Med..

[6] Campbell W.W., Evans W.J. (1996). Protein requirements of elderly people. Eur. J. Clin. Nutr..

[7] Kurpad A.V., Vaz M. (2000). Protein and amino acid requirements in the elderly. Eur. J. Clin. Nutr..

[8] Nowson C., O’Connell S. (2015). Protein requirements and recommendations for older people: A review. Nutrients.

[9] Elango R., Ball R.O., Pencharz P.B. (2012). Recent advances in determining protein and amino acid requirements in humans. Br. J. Nutr..

[10] National Academy of Sciences, The Institute of Medicine, Food and Nutrition Board (2005). Dietary Reference Intakes: Energy, Carbohydrate, Fiber, Fat, Fatty Acids, Cholesterol, Protein and Amino Acids.

[11] Rand W.M., Pellett P.L., Young V.R. (2003). Meta-analysis of nitrogen balance studies for estimating protein requirements in healthy adults. Am. J. Clin. Nutr..

[12] Hegsted D.M. (1976). Editorial: Balance Studies. J. Nutr..

[13] Young V.R. (1986). Nutritional balance studies: Indicators of human requirements or of adaptive mechanisms?. J. Nutr..

[14] Scrimshaw N.S. (1996). Criteria for valid nitrogen balance measurement of protein requirements. Eur. J. Clin. Nutr..

[15] Waterlow J.C. (1999). The mysteries of nitrogen balance. Nutr. Res. Rev..

[16] Millward D.J. (2001). Methodological considerations. Proc. Nutr. Soc..

[17] Rand W.M., Young V.R., Scrimshaw N.S. (1976). Change of urinary nitrogen excretion in response to low-protein diets in adults. Am. J. Clin. Nutr..

[18] Young V.R., Taylor Y.S., Rand W.M., Scrimshaw N.S. (1973). Protein requirements of man: Efficiency of egg protein utilization at maintenance and submaintenance levels in young men. J. Nutr..

[19] Elango R., Humayun M.A., Ball R.O., Pencharz P.B. (2010). Evidence that protein requirements have been significantly underestimated. Curr. Opin. Clin. Nutr. Metab. Care.

[20] Humayun M.A., Elango R., Ball R.O., Pencharz P.B. (2007). Reevaluation of the protein requirement in young men with the indicator amino acid oxidation technique. Am. J. Clin. Nutr..

[21] Joint WHO, FAO, UNU Expert Consultation (2007). Protein and amino acid requirements in human nutrition. World Health Organ. Tech. Rep. Ser..

[22] Campbell W.W., Johnson C.A., Johnson C.A., Mccabe G.P., Carnell N.S. (2008). Dietary protein requirements of younger and older adults. Am. J. Clin. Nutr..

[23] Ball R.O., Bayley H.S. (1984). Tryptophan requirement of the 2.5-kg piglet determined by the oxidation of an indicator amino acid. J. Nutr..

[24] Elango R., Ball R.O., Pencharz P.B. (2008). Indicator amino acid oxidation: Concept and application. J. Nutr..

[25] Pencharz P.B., Ball R.O. (2003). Different approaches to define individual amino acid requirements. Ann. Rev. Nutr..

[26] Zello G.A., Wykes L.J., Ball R.O., Pencharz P.B. (1995). Recent advances in methods of assessing dietary amino acid requirements for adult humans. J. Nutr..

[27] Tang M., McCabe G.P., Elango R., Pencharz P.B., Ball R.O., Campbell W.W. (2014). Assessment of protein requirement in octogenarian women with use of the indicator amino acid oxidation technique. Am. J. Clin. Nutr..

[28] Rafii M., Chapman K., Elango R., Campball W.W., Ball R.O., Pencharz P.B., Courtney-Maitin G. (2015). Dietary protein requirement of female adults >65 years determined by the indicator amino acid oxidation technique is higher than current recommendations. J. Nutr..

[29] Rafii M., Chapman K., Elango R., Campball W.W., Ball R.O., Pencharz P.B., Courtney-Maitin G. (2016). Dietary protein requirement of men >65 years old determined by the indicator amino acid oxidation technique is higher than the current estimated average requirement. J. Nutr..

[30] Morais J.A., Ross R., Pencharz P.B., Jones P.J., Ross R., Marliss E.B. (2000). Distribution of protein turnover changes with age in humans as assessed by whole-body magnetic resonance image analysis to quantify tissue volumes. J. Nutr..

[31] Morais J.A., Gougeon R., Pencharz P.B., Jones P.J., Ross R., Marliss E.B. (1997). Whole-body protein turnover in the healthy elderly. Am. J. Clin. Nutr..

[32] Janssen I., Heymsfield S.B., Ross R. (2002). Low relative skeletal muscle mass (sarcopenia) in older persons is associated with functional impairment and physical disability. J. Am. Geriatr. Soc..

[33] Sekhar R.V., Patel S.G., Guthikonda A.P., Reid M., Balasubramanyam A., Taffet G.E., Jahoor F. (2011). Deficient synthesis of glutathione underlies oxidative stress in aging and can be corrected by dietary cysteine and glycine supplementation. Am. J. Clin. Nutr..

[34] Johannsen D.L., Conley K.E., Bajpeyi S., Punyanitya M., Gallagher D., Zhang Z.Y., Covington J., Smith S.R., Ravussin E. (2012). Ectopic lipid accumulation and reduced glucose tolerance in elderly adults are accompanied by altered skeletal muscle mitochondrial activity. J. Clin. Endocrinol. Metab..

[35] Moore D.R., Churchward-Venne T.A., Witard O., Breen L., Burd N.A., Tipton K.D., Phillips S.M. (2014). Protein ingestion to stimulate myofibrillar protein synthesis requires greater relative protein intakes in healthy older versus younger men. J. Gerontol. A Biol. Sci. Med. Sci..

[36] Katsanos C.S., Kobayashi H., Sheffield-Moore M., Aarsland A., Wolfe R.R. (2006). A high proportion of leucine is required for optimal stimulation of the rate of muscle protein synthesis by essential amino acids in the elderly. Am. J. Physiol. Endocrinol. Metab..

[37] Volpi E., Mittendorfer B., Wolf S.E., Wolfe R.R. (1999). Oral amino acids stimulate muscle protein anabolism in the elderly despite higher first-pass splanchnic extraction. Am. J. Physiol..

[38] Volpi E., Kobayashi H., Sheffield-Moore M., Mittendorfer B., Wolfe R.R. (2003). Essential amino acids are primarily responsible for the amino acid stimulation of muscle protein anabolism in healthy elderly adults. Am. J. Clin. Nutr..

[39] Zanchi N.E., Nicastro H., Lancha A.H. (2008). Potential antiproteolytic effects of L-leucine: Observations of in vitro and in vivo studies. Nutr. Metab. (Lond.).

[40] Volpi E., Sheffield-Moore M., Rasmussen B.B., Wolfe R.R. (2001). Basal muscle amino acid kinetics and protein synthesis in healthy young and older men. JAMA.

[41] Symons T.B., Schutzler S.E., Cocke T.L., Chinkes D.L., Wolfe R.R., Paddon-Jones D. (2007). Aging does not impair the anabolic response to a protein-rich meal. Am. J. Clin. Nutr..

[42] Symons T.B., Sheffield-Moore M., Wolfe R.R., Paddon-Jones D. (2009). A moderate serving of high-quality protein maximally stimulates skeletal muscle protein synthesis in young and elderly subjects. J. Am. Diet. Assoc..

[43] Paddon-Jones D., Sheffield-Moore M., Zhang X.J., Volpi E., Wolf S.E., Aarsland A., Ferrando A.A., Wolfe R.R. (2004). Amino acid ingestion improves muscle protein synthesis in the young and elderly. Am. J. Physiol. Endocrinol. Metab..

[44] Katsanos C.S., Kobayashi H., Sheffield-Moore M., Aarsland A., Wolfe R.R. (2005). Aging is associated with diminished accretion of muscle proteins after the ingestion of a small bolus of essential amino acids. Am. J. Clin. Nutr..

[45] Volpi E., Mittendorfer B., Rasmussen B.B., Wolfe R.R. (2000). The response of muscle protein anabolism to combined hyperaminoacidemia and glucose-induced hyperinsulinemia is impaired in the elderly. J. Clin. Endocrinol. Metab..

[46] Paddon-Jones D., Rasmussen B.B. (2009). Dietary protein recommendations and the prevention of sarcopenia. Curr. Opin. Clin. Nutr. Metab. Care.

[47] Bouillanne O., Curis E., Hamon-Vilcot B., Nicolise I., Chrétien P., Schauer N., Vincent J.P., Cynober L., Aussel C. (2013). Impact of protein pulse feeding on lean mass in malnourished and at-risk hospitalized elderly patients: A randomized controlled trial. Clin. Nutr..

[48] Bouillanne O., Neveux N., Nicolis I., Curis E., Cynober L., Aussel C. (2014). Long-lasting improved amino acid bioavailability associated with protein pulse feeding in hospitalized elderly patients: A randomized controlled trial. Nutrition.

[49] Pennings B., Groen B., de Lange A., de Lange A., Gijsen A.P., Zorenc A.H., Senden J.M.G., van Loon L.J.C. (2012). Amino acid absorption and subsequent muscle protein accretion following graded intakes of whey protein in elderly men. Am. J. Physiol. Endocrinol. Metab..

[50] Gorissen S.H.M., Burd N.A.B., Hamer H.M., Gijsen A.P., Groen B.B., van Loon L.J.C. (2014). Carbohydrate coingestion delays dietary protein digestion and absorption but does not modulate postprandial muscle protein accretion. J. Clin Endocrinol. Metab..

[51] Loenneke J.P., Loprinzi P.D., Murphy C.H., Phillips S.M. (2016). Per meal dose and frequency of protein consumption is associated with lean mass and muscle performance. Clin. Nutr..

[52] Houston D.K., Nicklas B.J., Ding J.Z., Harris T.B., Tylavsky F.A., Newman A.B., Lee J.S., Sahyoun N.R., Visser M., Kritchevsky S.V. (2008). Dietary protein intake is associated with lean mass change in older, community-dwelling adults: The Health, Aging, and Body Composition (Health ABC) Study. Am. J. Clin. Nutr..

[53] Sahni S., Mangano K.M., Hannan M.T., Kiel D.P., Mclean R.R. (2015). Higher Protein intake is associated with higher lean mass and quadriceps muscle strength in adult men and women. J. Nutr..

[54] Tieland M., Dirks M.L., van der Zwaluw N., Verdijk L.B., van de Rest O., de Groot L.C.P.G.M., van Loon L.J.C. (2012). Protein supplementation increases muscle mass gain during prolonged resistance-type exercise training in frail elderly people: A randomized, double-blind, placebo-controlled trial. J. Am. Med. Dir. Assoc..

[55] Tieland M., van de Rest O., Dirks M.L., van der Zwalum N., Mensink M., van Loon L.J.C., de Groot L.C.P.G.M. (2012). Protein supplementation improves physical performance in frail elderly people: A randomized, double-blind, placebo-controlled trial. J. Am. Med. Dir. Assoc..

[56] Darling A.L., Millward D.J., Torgerson D.J., Hewitt C.E., Lanham-New S.A. (2009). Dietary protein and bone health: A systematic review and meta-analysis. Am. J. Clin. Nutr..

[57] Sahni S., Cupples L.A., Mclean R.R., Tucher K.L., Broe K.E., Kiel D.P., Hannan M.T. (2010). Protective effect of high protein and calcium intake on the risk of hip fracture in the Framingham offspring cohort. J. Bone Min. Res..

[58] Mangano K.M., Sahni S., Kerstetter J.E. (2014). Dietary protein is beneficial to bone health under conditions of adequate calcium intake: An update on clinical research. Curr. Opin. Clin. Nutr. Metab. Care.

[59] Gaffney-Stomberg E., Insogna K.L., Rodriguez N.R., Kerstetter J.E. (2009). Increasing dietary protein requirements in elderly people for optimal muscle and bone health. J. Am. Geriatr. Soc..

[60] Friedman A.N. (2004). High-protein diets: Potential effects on the kidney in renal health and disease. Am. J. Kidney Dis..

[61] Martin W.F., Armstrong L.E., Rodriguez N.R. (2005). Dietary protein intake and renal function. Nutr. Metab. (Lond.).

[62] Nguyen D., Samson S.L., Reddy V.T., Gonzalez E.V. (2013). Impaired mitochondrial fatty acid oxidation and insulin resistance in aging: Novel protective role of glutathione. Aging Cell.

[63] Jackson A.A., Gibson N.R., Lu Y., Jahoor F. (2004). Synthesis of erythrocyte glutathione in healthy adults consuming the safe amount of dietary protein. Am. J. Clin. Nutr..

[64] Courtney-Martin G., Rafii M., Wykes L.J., Ball R.O., Pencharz P.B. (2008). Methionine-adequate cysteine-free diet does not limit erythrocyte glutathione synthesis in young healthy adult men. J. Nutr..

